# Endometrium and endometriosis tissue mitochondrial energy metabolism in a nonhuman primate model

**DOI:** 10.1186/s12958-019-0513-8

**Published:** 2019-08-24

**Authors:** Hannah M. Atkins, Manish S. Bharadwaj, Anderson O’Brien Cox, Cristina M. Furdui, Susan E. Appt, David L. Caudell

**Affiliations:** 1 Department of Pathology, Section on Comparative Medicine, Wake Forest School of Medicine, Winston-Salem, One Medical Center Blvd, Winston-Salem, NC 27157 USA; 2 Department of Internal Medicine, Section on Molecular Medicine, Wake Forest School of Medicine, Winston-Salem, NC USA; 3 Agilent Technologies, Cell Analysis Division, Lexington, MA USA

**Keywords:** Infertility, Metabolism, Mitochondrial function, Nonhuman primate, Endometriosis

## Abstract

**Background:**

Endometriosis is the growth of uterine lining (endometrium) outside of the uterus. In other chronic inflammatory diseases, mitochondrial dysfunction is suspected of playing a role in disease pathogenesis. However, little is known about endometriosis mitochondrial function or its effects on tissue metabolism. The objectives of this study were to analyze mitochondrial function in nonhuman primate (NHP) endometrium and endometriosis tissue and to identify the metabolic features of these tissues that may contribute to disease.

**Methods:**

Mitochondrial function in endometriosis tissue and endometrium was measured using mitochondrial respirometry analysis to determine if changes in oxidative phosphorylation exist in endometrium and endometriosis tissue compared to control endometrium from clinically healthy NHPs. Targeted metabolomics and multidimensional statistical analysis were applied to quantify key metabolites in energy and amino acid biosynthesis pathways.

**Results:**

Mitochondrial respirometry assays showed endometrium from NHPs with endometriosis had reduced complex II-mediated oxygen consumption rates (OCR) across all energy states (basal, *p* = 0.01; state 3, *p* = 0.02; state 3u, *p* = 0.04; state 4o, *p* = 0.008) and endometriosis tissue had reduced state 3, complex I-mediated OCR (*p* = 0.02) and respiratory control rates (*p* = 0.01) compared to normal endometrium. Targeted metabolomics performed on tissue revealed carnitine (*p* = 0.001), creatine phosphate (*p* = 0.01), NADH (*p* = 0.0001), FAD (*p* = 0.001), tryptophan (*p* = 0.0009), and malic acid (*p* = 0.005) were decreased in endometriosis tissue compared to normal endometrium samples. FAD (*p* = 0.004), tryptophan (*p* = 0.0004) and malic acid (*p* = 0.03) were significantly decreased in endometrium from NHPs with endometriosis compared to normal endometrium. Significant metabolites identified in endometriosis and endometrium samples from animals with endometriosis were part of amino acid biosynthesis or energy metabolism pathways.

**Conclusions:**

Here, endometrial mitochondrial energy production and metabolism were decreased in endometrium and endometriosis tissue. Decreased mitochondrial energy production may be due to oxidative stress-induced damage to mitochondrial DNA or membranes, a shift in cell metabolism, or decreased energy substrate; however, the exact cause remains unknown. Additional research is needed to determine the implications of reduced mitochondrial energy production and metabolism on endometriosis and endometrium.

**Electronic supplementary material:**

The online version of this article (10.1186/s12958-019-0513-8) contains supplementary material, which is available to authorized users.

## Background

Endometriosis is a chronic gynecological disease characterized by oxidative stress and chronic inflammation [1, 2]. Macrophages, and to a lesser extent lymphocytes, produce reactive oxygen species (ROS) in an attempt to clear the endometriosis tissue and scavenge hemoglobin [3]. Hemoglobin-bound iron from inflammation-induced erythrocyte diapedesis or cyclic endometrium-like tissue sloughing is another source of ROS in endometriosis tissue [3–5]. Studies of endometriosis and other chronic inflammatory diseases (e.g., Crohn’s disease) suggest oxidative stress may also originate from other metabolic sources, such as defects in mitochondrial metabolism [1, 6]. Mechanistically, epithelial cell cancers that seed the abdomen in a similar manner to endometriosis (i.e., ovarian cancer), overwhelmingly support mitochondrial metabolism as a factor contributing to the persistence of cancer cells within the abdomen and is significantly altered compared to non-cancerous, eutopic cell analogs [7, 8]. There is a paucity of information in the current literature on endometriosis or endometrial mitochondrial function and how mitochondrial function may contribute to endometriosis-associated oxidative stress. Direct evaluation of endometriosis and endometrial mitochondrial function may provide insight into cellular metabolism and disease pathogenesis. Furthermore, mitochondrial ROS could be a potential therapeutic target for decreasing oxidative stress in endometriosis, which has shown to attenuate disease progression in preclinical animal models [9].

The metabolome of a biosystem may provide insight into changes in mitochondrial function or ROS production. Few metabolomics-based studies have investigated metabolic alterations in endometriosis. According to those studies, women with endometriosis had elevated serum levels of 11 metabolites involved in pyruvate metabolism and oxidative stress [1, 10]. Previous studies also identified a diagnostic pattern of serum metabolites in women with endometriosis that had a predictive ability greater than 80% and sensitivity, specificity, and classification rates higher than 90% [1, 10]. Despite the patterns observed in serum metabolites from women with endometriosis, these studies do not provide much information on tissue metabolism as serum metabolites may not mirror concentrations in tissue [11, 12]. Therefore, we used a nonhuman primate (NHP) model of spontaneous endometriosis in combination with mitochondrial respirometry assays, and targeted metabolomics to determine metabolic alterations in endometrium and endometriosis tissue compared to endometrium from clinically healthy controls. NHP tissues taken at necropsy provide a highly translatable platform for studying endometrial metabolism. In addition, NHPs provide a source of clinically healthy endometrium without the ethical issues involved in endometrial biopsy of clinically healthy women. We hypothesized that endometriosis tissue would have increased mitochondrial respiration due to the relative proliferative nature of the tissue [13]. Conversely, we hypothesized endometrium would have decreased respiration similar to other tissues affected by ROS [6]. We also anticipated elevated tissue concentrations of metabolites related to oxidative stress and pyruvate metabolism in both endometriosis and endometrium and that these changes would mirror those previously observed in human serum [1, 10, 14]. This study provides an opportunity to better understand endometriosis pathogenesis and the effects of metabolism on endometrial biology. In addition, treating chronic inflammation or mitochondrial dysfunction in endometriosis may be a strategy to reduce metabolic biomarkers of systemic oxidative stress and effects of inflammation on the endometrium. Ultimately, decreasing associated chronic inflammation may improve overall systemic health in women with endometriosis [15–17].

## Methods

Seventeen macaque nonhuman primates (NHP, *Macaca fascicularis* and *M. mulatta)* with endometriosis and eight NHPs without endometriosis were identified using the Wake Forest School of Medicine, Primate Center Necropsy Database (total NHP, *n* = 25). Endometrium (EM) and endometriosis (EMO) tissues were harvested from the NHPs at necropsy as part of the experimental endpoint of other studies and were used immediately after necropsy (mitochondrial function study) or flash frozen and stored at − 80 °C (metabolomics study). Endometriosis cases were assigned a modified American Society for Reproductive Medicine (ASRM) classification of endometriosis severity [18, 19]. Endometrium (nEM) was similarly collected and stored from a subset of the clinically healthy animals. Similar to endometriosis studies in humans, the NHPs consumed a variety of diets including high-fat typical Western diets and plant-based monkey chow. There was no significant difference in the number of monkeys receiving the various diets. All animal studies were approved by the Wake Forest School of Medicine (WFSM) IACUC, using the Guide for the Care and Use of Animals and the Welfare Act and Animal Welfare Regulations [20, 21]. WFSM is accredited by the Association for Assessment and Accreditation of Laboratory Animal Care International (AAALAC). The tissue samples were then used for the mitochondrial bioenergetics and metabolomics studies described below.

### Mitochondrial bioenergetics

The mitochondrion is the cell’s power plant, which provides an efficient transfer of electrons from the reduced forms of nicotinamide adenine dinucleotide (NADH) and flavin adenine dinucleotide (FADH_2_) to oxygen and, eventually, the high-energy phosphate bond of adenosine triphosphate (ATP) [22]. A high energy electron is transferred among membrane complexes I (NADH dehydrogenase complex), III (cytochrome b-c_1_ complex), and IV (cytochrome oxidase complex), which moves protons into the space between the inner and outer mitochondrial membranes (i.e., the intermembrane space) [23]. Proton movement into the intermembrane space is responsible for formation and maintenance of an electrical voltage gradient across the inner mitochondrial membrane, driving ATP production via ATP synthase or complex V. Complex V uses ion flux from the inner membrane space to the mitochondrial matrix to catalyze ATP synthesis from ADP, a proton, and inorganic phosphate. One method of analyzing ETC function in tissue is through mitochondrial isolation and evaluation of oxygen consumption during five energy states: baseline (state 2); or after the addition of adenosine diphosphate (ADP, state 3); oligomycin (state 4o); carbonyl cyanide p-(tri-fluromethoxy) phenyl-hydrazone (FCCP, state 3u); or antimycin A [24]. Addition of ADP to the assay measures maximum oxidative phosphorylation by providing ADP as an energy substrate. Oligomycin inhibits ATP synthase (complex V) and therefore shuts down the production of ATP from ADP. FCCP allows protons to rush through the inner mitochondrial membrane into the matrix, thereby “uncoupling” the rate of ETC progression and ATP synthesis. Finally, antimycin A blocks the transfer of electrons at protein complex III and shuts down the energy transfer and proton gradient needed for ATP production. In addition to overall mitochondrial oxidative phosphorylation, specific proteins in the electron transport chain can be assessed with the addition of specific substrates or ETC protein blocking agents.

Mitochondria were isolated from EMO (*n* = 4) and EM (*n* = 4) from four NHPs with endometriosis (age 11.5–16 years, mean 14.6 years) and nEM from five clinically healthy NHPs (ages 12–14 years, mean 12.8 years) (Additional file 1: Figure S1). The nine animals were a subset of the 25 total NHPs described above. EM and EMO Complex-II mediated respiration data from one NHP with endometriosis was not usable nEM, resulting in *n* = 3 for those tests. EM, and EMO were placed in Dulbecco’s phosphate-buffered saline on wet ice and mitochondrial isolation initiated immediately following necropsy. Briefly, whole tissues were homogenized and mitochondria separated from the rest of the cell pellet by density gradient centrifugation [25]. The isolated mitochondrial oxygen consumption rate (OCR) was measured in duplicate after adding ADP, oligomycin, FCCP, or antimycin A using the Seahorse XF24–3 Extracellular Flux Analyzer (Agilent Technologies, Santa Clara, CA, USA). Both five and ten micrograms (μg) of mitochondrial protein were tested in the analyzer to determine optimal conditions. All samples were run in duplicate. Mitochondrial oxygen consumption was reported as picomole of oxygen per μg of mitochondrial protein. Assays specifically examined the function of electron transport chain complex II-mediated respiration, (with the addition of succinate as substrate and rotenone to block complex I) and complex I-mediated respiration (with the addition of pyruvate as the substrate and malonate to block complex II) (Additional file 2: Figure S2). Respiratory control rate (RCR) was calculated for each group and complex (I or II) as OCR from State 3u/State 4o. Proton leak was calculated as the difference between State 4o and post-antimycin A OCR [24]. OCR, RCR, and proton leak for each complex were compared using one-way analysis of variance (ANOVA). *P* values less than 0.05 were considered significant.

In addition to endometriosis severity, samples were analyzed for mitochondrial function according to the phase of the menstrual cycle (follicular or luteal phase). Within the normal group, three animals were in the luteal phase, and one was in the follicular phase at the time of tissue collection. In the endometriosis group, two animals were in luteal phase, one was in follicular phase, and one had histologically atrophic endometrium. When combined, regardless of disease status there were five NHPs in the luteal phase, two in the follicular phase and one with histologically atrophic endometrium. Menstrual cycle phase was determined by a combination of histopathological evaluation, daily vaginal swab records, or serum estrogen to progesterone ratios. The OCR, RCR, and proton leak was determined for each menstrual cycle phase and analyzed using a one-way ANOVA. *P* values less than 0.05 were considered significant.

### Targeted Metabolomic analysis

From the initial 25 NHPs identified through the Wake Forest School of Medicine Primate Center Necropsy Database, eight nEM samples, 12 EM samples, and 17 EMO samples were analyzed for 28 randomly selected metabolites (Table 1) by the Wake Forest Proteomics and Metabolomics Shared Resource facility (Additional file 3: Figure S3). Five endometrial samples from the NHPs with endometriosis were not available for analysis due to distortion of the uterus from endometriosis or inadequate volume. For metabolite extraction, a portion of each tissue sample was weighed into a homogenization tube containing 1.4 μm ceramic beads. After weighing, 800 μL of methanol, 250 μL of water, and 50 μL of 500 ng/μL 2-(N-morpholino) ethanesulfonic acid (MES) internal standard solution were added to the tubes. The tubes were then closed and homogenized with a Bead Ruptor 24 (OMNI International, Kennesaw, GA) in 6 cycles of 20 s. The contents were then transferred to glass tubes to which 800 μL of chloroform and 400 μL of water were added. The tubes were then mixed thoroughly and centrifuged to separate the layers. The top layer of each extract was then collected and evaporated to dryness in a SpeedVac evaporator. Each sample was then reconstituted in 1 mL of water and centrifuged at 18,000 x g for 10 min. The supernatants were collected for LC-MS/MS analysis.

**Table 1:** Targeted metabolites

Glutathione	Adenosine	Valine
Nicotinamide adenine dinucleotide (NADH)	Flavin adenine dinucleotide (FAD)	Methionine
Glycine	Phenylalanine	Fumaric Acid
Aspartic acid	Tryptophan	Citric Acid
Glutamine	Glucose 6-phosphate	Lactic Acid
Lysine	Glutamic acid	Uracil
Threonine	Alanine	Carnitine
L-α-Glycerophosphocholine	Histidine	Malic Acid
Nicotinic acid	Arginine	
Leucine	Creatine Phosphate	

LC-MS/MS analysis was performed using a Shimadzu UHPLC-MS/MS equipped with two LC-30 AD pumps, a SIL-30 AC autosampler, a CBM-20A communications bus module, a DGU-20A5R degassing unit, a CTO-30A column oven, and an 8050 triple-quadrupole mass spectrometer utilizing a DUIS source. The analytical samples underwent separation on a Restek Ultra PFPP column (150 × 2.1 mm, 3 μm; Restek, Bellefonte, PA) under gradient conditions with a mobile phase A consisting of 0.1% formic acid in water and a mobile phase B consisting of 0.1% formic acid in 100% acetonitrile. The gradient began with 2 min at 0% B followed by a ramp to 25% B between 2 and 5 min, another ramp to 35% B between 5 and 11 min, a final increase from 35 to 95% B between 11 and 15 min, a hold at 95% B from 15 to 20 min, then a decrease to 0% B between 20 and 20.10 min, and a final hold at 0% B from 20.10 to 25 min. A flow rate of 0.25 mL/minute was used with a sample injection volume of 10 μL. Ionization in positive and negative ESI modes occurred in the DUIS source with the following conditions: nebulizing gas flow of 2 L/min, heating gas flow of 5 L/min, interface temperature of 350 °C, DL temperature of 250 °C, heat block temperature of 400 °C, and a drying gas flow of 15 L/min. Additional mass spectroscopy settings are provided in Additional file 5: Table S1.

Statistical analyses of tissue metabolite concentrations were completed using MetaboAnalyst 4.0 (http://www.metaboanalyst.ca) [26–28]. Briefly, concentrations were log-transformed to normalize the data. Next, principal component analysis (PCA) and partial least squares discriminate analysis (PLS-DA) were used to identify possible associations among metabolites and predictive ability of metabolites to determine differences in tissue type (nEM, EM, or EMO). Metabolites were also analyzed using variable importance in projection (VIP) scores [29]. Metabolites with PLS-DA VIP scores above one were considered potentially significant and further analyzed using one-way ANOVA and Tukey’s HSD post-doc tests to determine significant metabolic differences between EM or EMO and nEM. *P* values less than 0.05 were considered significant. Significant metabolites were entered into the Kyoto Encyclopedia of Genes and Genomes (KEGG) pathway analysis in MetaboAnalyst 4.0 to determine possible involved pathways. The top ten matched pathways for all metabolites identified through MetaboAnalyst 4.0 were 1) aminoacyl-tRNA biosynthesis, 2) nitrogen metabolism, 3) alanine, aspartate, and glutamate metabolism, 4) arginine and proline metabolism, 5) glycine, serine, and threonine metabolism, 6) pantothenate and CoA biosynthesis, 7) valine, leucine and isoleucine biosynthesis, 8) cysteine and methionine metabolism, and 9) cysteine and methionine metabolism, and 10) citric acid cycle. Pre-matching all targeted metabolites to pathways allows for comparison between possible pathway combinations and those identified after analysis.

## Results

### Mitochondrial Respirometry

**Fig. 1:**
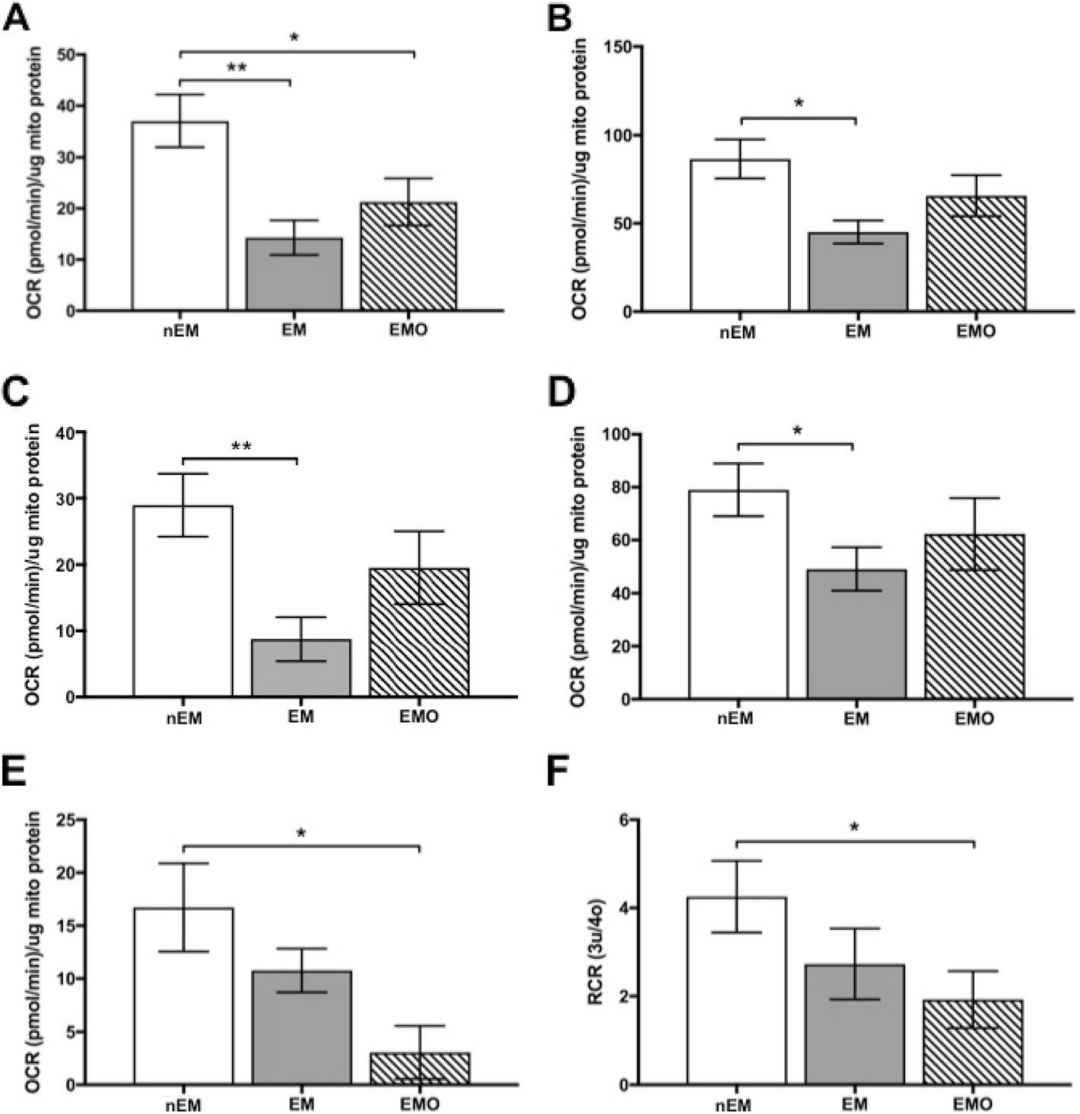
Complex II and I-mediated respiration are decreased in endometrium and endometriosis tissue from NHPs with endometriosis. **a**) Endometrium (EM, *p* = 0.003) and endometriosis (EMO, *p* = 0.04) tissue mitochondrial OCR was decreased at complex II-mediated basal respiration compared to normal endometrium (nEM). **b**) Complex II-mediated State 3 (*p* = 0.02), **c**) State 4o (*p* = 0.008), and **d**) State 3u (*p* = 0.04) respiration was decreased in endometrium from NHPs with endometriosis. One-way ANOVA, *p* < 0.05 considered significant. **e**) State 3 complex I-mediated OCR (*p* = 0.02) and **f**) complex I respiratory control rate (RCR, *p* = 0.01) was decreased in EMO compared to nEM. One-way ANOVA, *p* < 0.05 was considered significant. Sample sizes were nEM, *n* = 4; EM, *n* = 3; EMO, *n* = 3 for A-D and nEM, *n* = 5; EM, *n* = 4; EMO, *n* = 4 with multiple measurements per energy state for E and F

Electron transport chain complex I- and II-mediated mitochondrial respiration was decreased in EM and EMO. Specifically, EM had statistically significant decreased complex-II mediated OCR at baseline (Fig. 1a, *p* = 0.003) and in states 3 (Fig. 1b, *p* = 0.02), 4o (Fig. 1c, *p* = 0.008), and 3u (Fig. 1d, *p* = 0.04). EMO also had decreased complex II-mediated OCR in baseline energy state (Fig. 1a, *p* = 0.04). Furthermore, EMO also had decreased complex I-mediated respiration in state 3 (Fig. 1e, *p* = 0.02) and complex I RCR compared to nEM (Fig. 1e, *p* = 0.01). There was no difference in complex I- or II-mediated respiration RCR or protein leak among nEM, EM or EMO tissues.

Age and phase of the menstrual cycle were hypothesized to affect mitochondrial respiration. There was no significant difference in age between normal and endometriosis NHP cohorts (*p* = 0.12). Menstrual cycle phases were distributed equally between normal and endometriosis NHP cohorts except for one NHP with histologically atrophic EM in the endometriosis group, which was excluded from the cycle phase analyses. There was no difference in complex II- or complex I-mediated OCR, RCR, or proton leak when analyzed by menstrual cycle phase, combining animals with and without endometriosis. ASRM stages for the four NHPs with endometriosis were *n* = 1 with stage II and *n* = 3 with stage IV. Due to the limited number of stages represented in the endometriosis group, mitochondrial function was not analyzed according to ASRM stage.

### Endometriosis energy metabolism

**Fig. 2:**
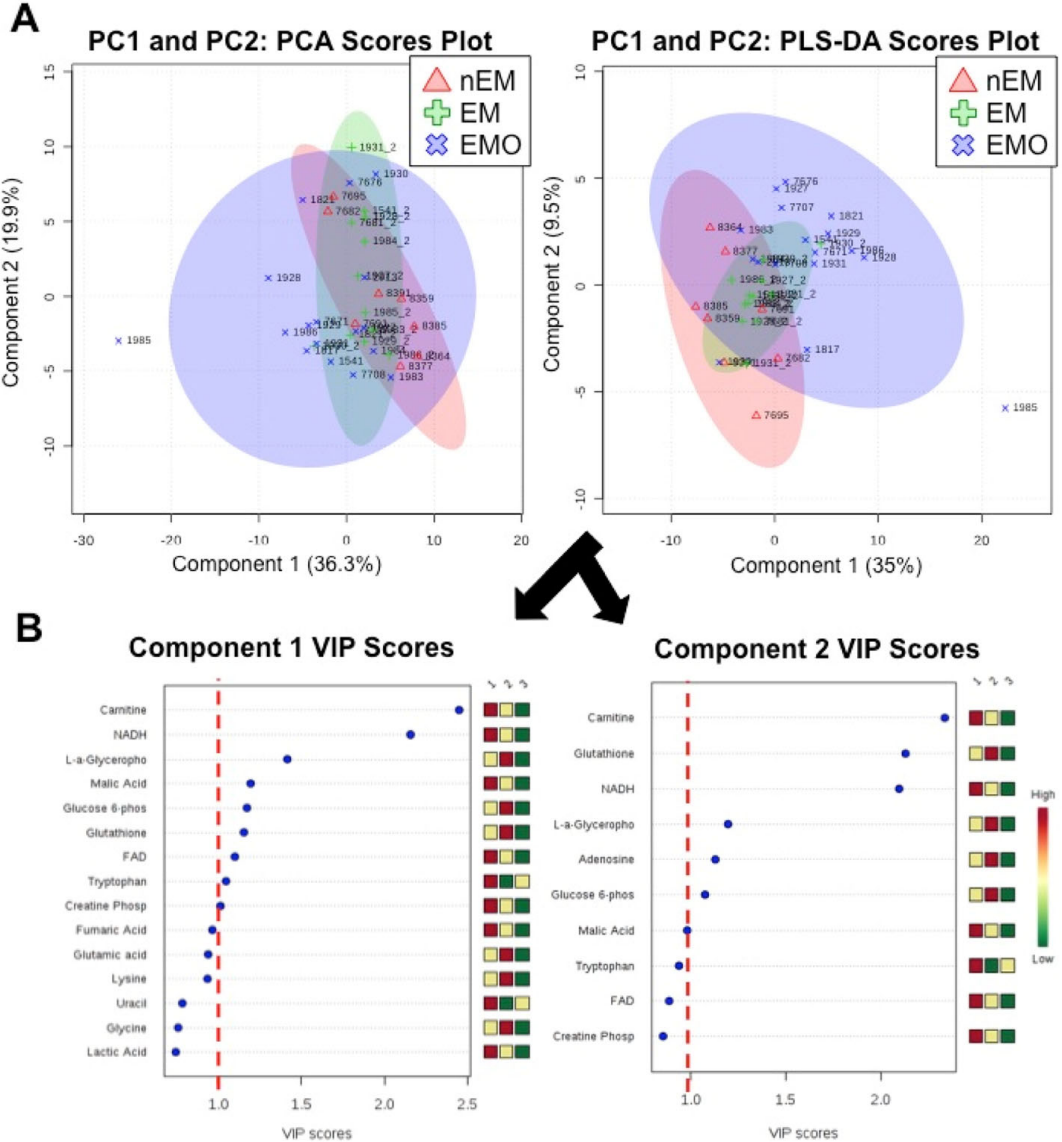
Significant metabolites were identified using principal component (PCA) and partial least squares (PLS-DA) scatter plots with variable importance in projection (VIP) scores. **a**) PCA and PLS-DA scatter plots, Red triangles are normal endometrium (nEM), green pluses are endometrium from NHPs with endometriosis (EM) and blue “x” represent endometriosis tissue (EMO). **b**) PLS-DA VIP scores for principle components 1 and 2. Metabolites with VIP scores equal to or greater than one were considered significant following guidelines set by Akarachantachote et al., 2014

PCA and PLS-DA did not identify distinct metabolic patterns among control, endometrium and endometriosis tissues (Fig. 2a). However, PLS-DA VIP scores identified ten significant metabolites based upon principal components one and two (Fig. 2b). These metabolites included: carnitine, nicotinamide adenine dinucleotide (NADH), L-α-glycerophosphocholine, malic acid, glucose-6-phosphate, glutathione, adenosine, creatine phosphate, tryptophan, and flavin adenine dinucleotide (FAD). One-way ANOVA confirmed significant differences among tissue types for six of the metabolites.

**Fig. 3:**
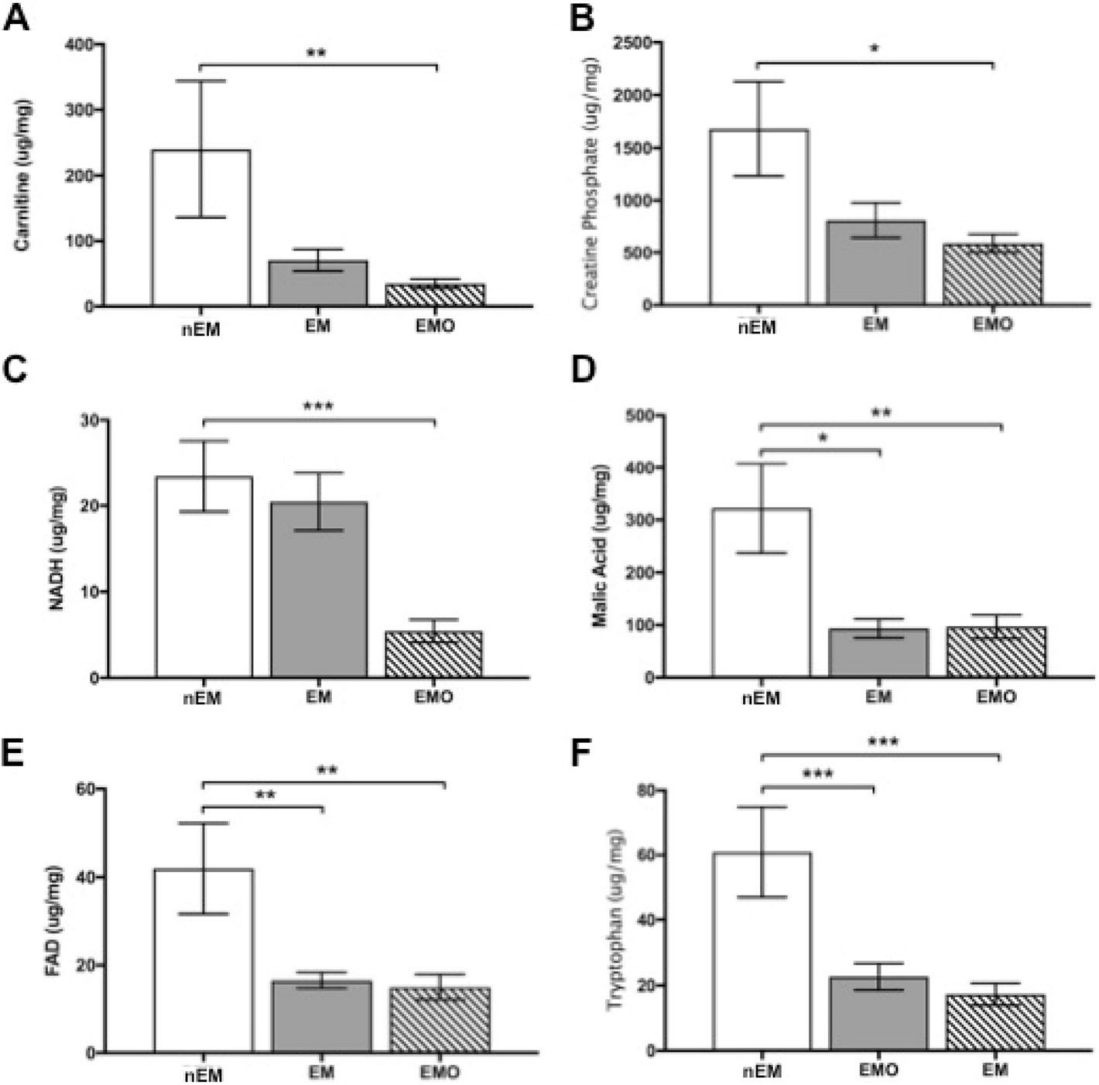
Metabolites in the amino acid biosynthesis, and riboflavin and nitrogen metabolism pathways are decreased in endometriosis tissue and endometrium. **a**) Carnitine (*p* = 0.002) and **b**) creatine phosphate (*p* = 0.01), were decreased in endometriosis tissue (EMO) compared to endometrium from clinically healthy nonhuman primates (NHPs) (nEM). **c**) Nicotinamide adenine dinucleotide (NADH) was decreased in EMO compared to nEM (*p* = 0.0001). **d**) Malic acid, was decreased in EMO (*p* = 0.005) and endometrium from NHPs with endometriosis (EM) (*p* = 0.03). **e**) Flavin adenine dinucleotide (FAD) and **f**) tryptophan concentrations were also decreased in EMO (FAD, *p* = 0.001; tryptophan, *p* = 0.0009) and EM (FAD, *p* = 0.004; tryptophan, *p* = 0.0004) compared to nEM. One-way ANOVA, *p* < 0.05 was considered significant. Carnitine and NADH data were back-transformed from natural log and square root, respectively. For all graphs, sample sizes were nEM, *n* = 8; EM, *n* = 12; and EMO, *n* = 17

Significant metabolites differed by tissue when compared to normal endometrium. Endometriosis tissue had decreased carnitine (Fig. 3a, *p* = 0.002), creatine phosphate (Fig. 3b, *p* = 0.01), NADH (Fig. 3c, *p* = 0.0001), malic acid (Fig. 3d, *p* = 0.005), and FAD (Fig. 3e, *p* = 0.001) compared to normal endometrium. In endometrium from NHPs with endometriosis, malic acid (Fig. 3d, *p* = 0.03), FAD (Fig. 3e, *p* = 0.004) and tryptophan (Fig. 3f, *p* = 0.0009) were significantly decreased compared to normal endometrium.

Pathway analysis of significant metabolites in endometriosis or endometrium were mapped to relevant pathways and compared to the original number of metabolites evaluated in each pathway (Table 2). Significantly decreased metabolites in endometriosis matched to the riboflavin metabolism pathway (1 of 1 original riboflavin metabolites); however, the *p* value reported for pathway matching was equal to 0.05. Significant metabolites in endometrium from NHPs with endometriosis mapped to two pathways: (1) tryptophan biosynthesis and metabolism (1 of 2 original metabolites, *p* = 0.03), and (2) nitrogen metabolism (1 of 1 original metabolite, *p* = 0.04).

**Table 2:** Pathway analysis results

Pathway Name	*p*-value	-log(p)
Endometrium (EM) Metabolic Pathways		
Tryptophan Metabolism	0.03	3.4
Nitrogen Metabolism	0.04	3.0
Endometriosis (EMO) Metabolic Pathway		
Riboflavin metabolism	0.05	3.0

## Discussion

Endometriosis tissue and endometrium from NHPs with endometriosis had evidence of decreased mitochondrial respiration, and tissue metabolism compared to endometrium from clinically healthy NHPs. In endometriosis tissue, complex I-mediated mitochondrial respiration and RCR were decreased. All energy states of complex II-mediated mitochondrial respiration were decreased in endometrium from NHPs with endometriosis. Metabolomic analysis of endometriosis tissue and endometrium revealed depletion of metabolites involved in tryptophan, nitrogen and riboflavin metabolism. Altogether these data may signify cellular damage, a shift in endometrial and endometriosis metabolism from mitochondria-driven oxidative phosphorylation to alternative pathways, or mitochondrial dysfunction.

One possible explanation for diminished metabolite concentrations and mitochondrial oxygen consumption is mitochondrial damage by ROS from hemoglobin oxidation, inflammatory cells, and ETC, resulting in less efficient energy production from available substrates. These changes could be due to negative feedback from ROS or damage to mitochondrial DNA resulting in functional mutations [30]. Decreased energy substrates in endometriosis and endometrium may be due to the proliferative nature of the tissue or due to degradation by proteases associated with inflammation [13]. A second possible mechanism for decreased mitochondrial respiration involves shunting of glucose into aerobic glycolysis (i.e., the Warburg effect) to meet increased energy demands of endometriosis tissue, similar to cancer cell metabolism [31]. There is also evidence that mutations in proto-oncogenes such as phosphatase and tensin homolog deleted on chromosome 10 (PTEN) may contribute to the unrestricted growth of endometriosis tissue further increasing cellular energy demands [32]. However, we did not see an increase in lactic acid as would be expected if glycolysis was being shunted away from oxidative phosphorylation to fermentation. Additional investigation into Akt/PI3K activity and MYC expression are needed to confirm such an increase in aerobic glycolysis in endometriosis tissue.

Differences in the electron transport chain components implicated in decreased mitochondrial respiration between endometriosis (complex I) and endometrium (complex II) may be explained by variable sources and concentrations of ROS within each tissue or the overall tissue environment. Women with endometriosis have increased serum and peritoneal fluid oxidative stress reflected in higher levels of nitric oxide, hydrogen peroxide, hydroxyl radicals, superoxide ions, and free iron [33]. Endometriosis tissue ROS likely originate from a combination of hemoglobin oxidation, mitochondria production, and inflammation resulting in superoxide and hydroxyl ions [4]. Alternatively, the source of ROS in endometrium may be more restricted to lower concentrations of circulating ROS combined with the cyclic production of anti-inflammatory enzymes such as cyclooxygenases at the time of menses [34].

Six metabolites involved in tryptophan, riboflavin, and nitrogen metabolism were significantly decreased in endometriosis tissue and endometrium from NHPs with endometriosis. These metabolites did not map to similar glucose metabolism and oxidative stress pathways identified in human serum and peritoneal fluid metabolomics studies [1, 10]. This discrepancy is likely a function of comparing serum to tissue metabolites. Overall, low levels of amino acids may be an indication of depletion possibly due to inflammation or cellular consumption; however, the cause in this study remains unclear. One specific amino acid synthesis pathway is worth highlighting: tryptophan metabolism. In endometriosis, inflammation-induced catabolism of tryptophan is thought to permit immune tolerance of endometriosis implants [35]. Additional global metabolomic analyses of serum and tissue are needed to confirm these findings.

Age and menstrual cycle phase had marginal effects on mitochondrial function. One NHP with endometriosis had histologically atrophic endometrium. Histologically atrophic endometrium does not necessarily mean the endometrium was insensitive to estrogen or progesterone stimulation. This particular NHP had an ASRM severity score of IV, presumably stimulated by ovarian or endometriosis production of estrogen [36]. Excluding this NHP from data analysis had little effect on outcomes. Baseline (Additional file 4: Figure S4A, *p* = 0.02), state 3 (Additional file 4: Figure S4B, *p* = 0.03), and state 4o (Additional file 4: Figure S4C, *p* = 0.02) complex-II mediated OCR remained statistically decreased in endometrium from NHPs with endometriosis. The major difference observed was that state 3u complex II-mediated respiration in endometrium from NHPs with endometriosis was not significantly decreased compared to normal endometrium (Additional file 4: Figure S4D, *p* = 0.23) but it was significantly decreased compared to endometriosis tissue (*p* = 0.03). There was no difference in endometriosis tissue complex I state 3 mitochondrial respiration compared to normal endometrium but the difference trended towards significance (not shown, *p* = 0.058). It is important to note that the sample size for this study was small. Any changes in statistical significance after excluding data from this NHP could be due to a type II error due to insufficient power [37]. A larger, more comprehensive study would clarify these findings.

The data point for NHP 1985 on the PCA and PLS-DA plots (Fig. 2a) appears to be outside of the rest of the data. Before performing the multi-dimensional analyses, the data were log-transformed and evaluated for Gaussian distribution. The resulting data followed a normal distribution and thus were used in the analyses. Upon seeing the data point for 1985, we went back to the data to determine if the any of the EMO data points were considered outliers (i.e., outside of three times the interquartile range above and below the mean). No major outliers were identified and any variation among metabolites for 1985 was accepted as part of the natural range of the data.

There are a few aspects of this study that should be considered when interpreting these data. First, mitochondrial bioenergetics were performed on isolated mitochondria from all cell types within the tissue (i.e., stromal, glandular epithelial, and inflammatory cells). It is likely that endometriosis tissue contains a decreased proportion of one of these cell types that may be more metabolically active. Additional experiments are warranted to identify differences in mitochondrial function between cell types. Second, when comparing endometriosis to eutopic endometrium one must take into account the genetic and physiologic differences between the tissues (diseased vs. normal) [38]. However, endometrium is highly similar morphologically and physiologically to endometriosis tissue; therefore, comparing differences between the two may provide insight into endometriosis pathogenesis and treatment.

## Conclusions

Endometrium and endometriosis from NHPs with endometriosis had decreased energy metabolism, likely resulting from shifts in energy metabolism away from oxidative phosphorylation or increased endometriosis-associated oxidative stress. If inflammation is found to be the cause of these metabolic changes, these data may support the combined use of anti-inflammatory drugs with hormones for endometriosis treatment. However, further studies are needed to better define the relationships of endometriosis-associated inflammation, mitochondrial function, and tissue metabolism.

## Additional files

Additional file 1: **Figure S1.** Schematic illustrating experimental design for mitochondrial respirometric analyses. Tissues were collected from four nonhuman primates (NHPs) with and four without endometriosis (*n* = 8 total NHPs). These NHPs were part of the larger metabolomics cohort. Endometrium and endometriosis tissue were collected from the four NHPs with endometriosis and were designated EM and EMO, respectively. Normal endometrium the clinically healthy NHPs was abbreviated nEM. Mitochondria were isolated from whole tissue and oxygen consumption rate (OCR) of electron transport chain (ETC) complex I- and complex II-mediated oxidative phosphorylation was assessed. OCR data for EM and EMO were compared to nEM using a one-way analysis of variance (ANOVA). Data from endometrium samples (EM and nEM) were combined and analyzed by ANOVA according to menstrual cycle phase. (DOCX 109 kb)

Additional file 2: **Figure S2.** A representative OCR output graph from the Seahorse XF24–3 Extracellular Flux Analyzer (Agilent Technologies, Santa Clara, CA, USA). In this example, samples were run according to tissue type (endometrium, EM; or endometriosis, EMO) and animal number (1985 or 1986) at 5 μg of mitochondrial protein. Samples were run using both 5 and 10 μg total mitochondrial protein. (DOCX 71 kb)

Additional file 3: **Figure S3.** Targeted metabolomics experimental design and statistical analysis. Eight clinically healthy and 17 nonhuman primates (NHP) with endometriosis were identified through the Wake Forest Primate Center Comprehensive Animal Record System (CARS). Of the 17 NHPs with endometriosis, 12 had available endometrium for analysis. Endometrium from clinically healthy animals was designated nEM. Endometrium and endometriosis tissue from NHPs with endometriosis were EM and EMO, respectively. Targeted metabolomics was performed by the Wake Forest Proteomics and Metabolomics Shared Resource facility. Twenty-nine metabolites were identified using LC-MS/MS. Statistical analyses of tissue metabolite concentrations were completed using MetaboAnalyst 4.0. Metabolite concentrations were log-transformed and analyzed by partial least squares (PLS) and principle component (PCA) analyses. Significant metabolites determined with PLS and PCA were further analyzed using one-way analysis of variance (ANOVA) and GraphPad statistical software. Finally, metabolites that proved significant with both PLS/PCA and ANOVA were entered into pathway analysis algorithms associated with MetaboAnalyst 4.0 to identify relevant pathways. (DOCX 108 kb)

Additional file 4: **Figure S4.** Complex II-mediated OCR remained significantly decreased after exclusion of the NHP with an atrophic endometrium. A) Endometrial mitochondrial OCR is decreased at baseline (EM, *p* = 0.02), B) energy state 3 (*p* = 0.03), and C) energy state 4o (*p* = 0.02). D) EM complex II-mediated OCR was no longer significant compared to normal endometrium (nEM) in state 3u (*p* = 0.04) but was significantly decreased compared to endometriosis tissue (EMO, *p* = 0.03). One-way ANOVA or nonparametric equivalent, *p* < 0.05 considered significant. (DOCX 110 kb)

Additional file 5: **Table S1.** M/z Transitions and Collision Energies (PDF 53 kb)

## Data Availability

The datasets analyzed in this study are available from the corresponding author upon request.
