# Supplementary figures and images for: Endometrium and endometriosis tissue mitochondrial energy metabolism in a nonhuman primate model
Source: Reprod Biol Endocrinol. 2019 Aug 24;17:70. doi: 10.1186/s12958-019-0513-8 (PMC6708555; doi:10.1186/s12958-019-0513-8)

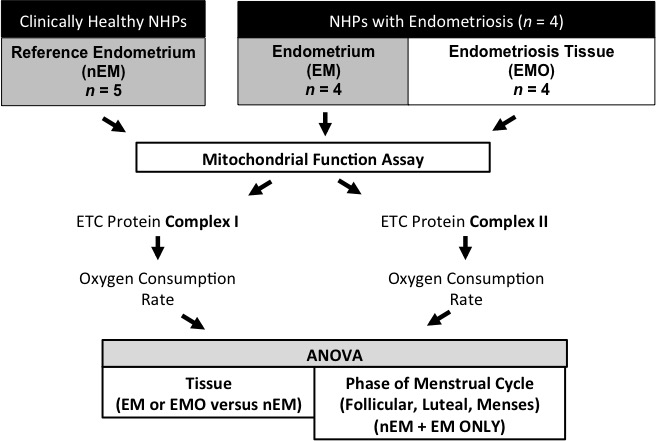

Supplement: Supplementary file 1 — Figure S1. Schematic illustrating experimental design for mitochondrial respirometric analyses. Tissues were collected from four nonhuman primates (NHPs) with and four without endometriosis (n = 8 total NHPs). These NHPs were part of the larger metabolomics cohort. Endometrium and endometriosis tissue were collected from the four NHPs with endometriosis and were designated EM and EMO, respectively. Normal endometrium the clinically healthy NHPs was abbreviated nEM. Mitochondria were isolated from whole tissue and oxygen consumption rate (OCR) of electron transport chain (ETC) complex I- and complex II-mediated oxidative phosphorylation was assessed. OCR data for EM and EMO were compared to nEM using a one-way analysis of variance (ANOVA). Data from endometrium samples (EM and nEM) were combined and analyzed by ANOVA according to menstrual cycle phase. (DOCX 109 kb) [file 12958_2019_513_MOESM1_ESM.docx]

**
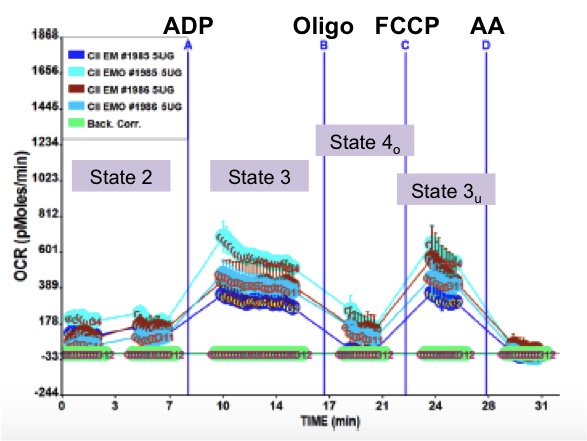
**

Supplement: Supplementary file 2 — Figure S2. A representative OCR output graph from the Seahorse XF24–3 Extracellular Flux Analyzer (Agilent Technologies, Santa Clara, CA, USA). In this example, samples were run according to tissue type (endometrium, EM; or endometriosis, EMO) and animal number (1985 or 1986) at 5 μg of mitochondrial protein. Samples were run using both 5 and 10 μg total mitochondrial protein. (DOCX 71 kb) [file 12958_2019_513_MOESM2_ESM.docx]

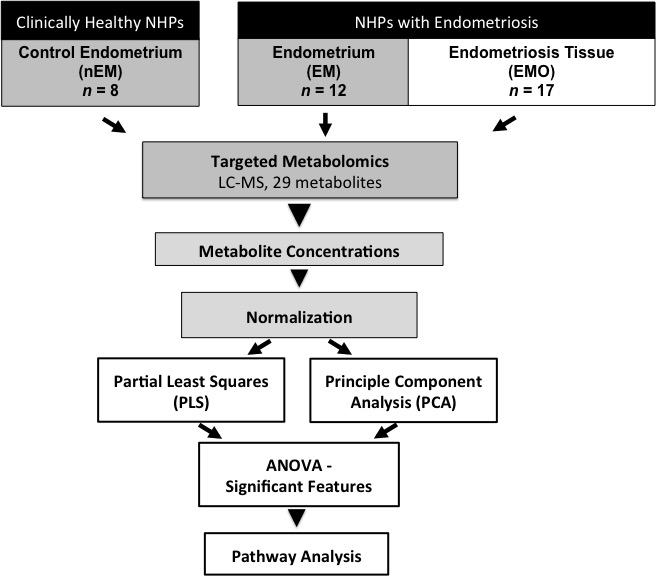

Supplement: Supplementary file 3 — Figure S3. Targeted metabolomics experimental design and statistical analysis. Eight clinically healthy and 17 nonhuman primates (NHP) with endometriosis were identified through the Wake Forest Primate Center Comprehensive Animal Record System (CARS). Of the 17 NHPs with endometriosis, 12 had available endometrium for analysis. Endometrium from clinically healthy animals was designated nEM. Endometrium and endometriosis tissue from NHPs with endometriosis were EM and EMO, respectively. Targeted metabolomics was performed by the Wake Forest Proteomics and Metabolomics Shared Resource facility. Twenty-nine metabolites were identified using LC-MS/MS. Statistical analyses of tissue metabolite concentrations were completed using MetaboAnalyst 4.0. Metabolite concentrations were log-transformed and analyzed by partial least squares (PLS) and principle component (PCA) analyses. Significant metabolites determined with PLS and PCA were further analyzed using one-way analysis of variance (ANOVA) and GraphPad statistical software. Finally, metabolites that proved significant with both PLS/PCA and ANOVA were entered into pathway analysis algorithms associated with MetaboAnalyst 4.0 to identify relevant pathways. (DOCX 108 kb) [file 12958_2019_513_MOESM3_ESM.docx]

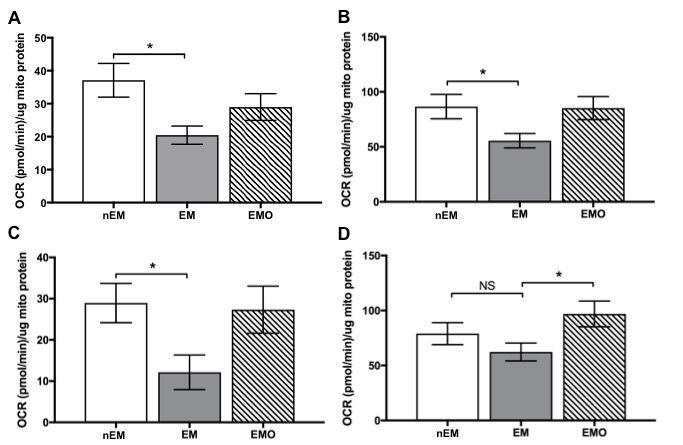

Supplement: Supplementary file 4 — Figure S4. Complex II-mediated OCR remained significantly decreased after exclusion of the NHP with an atrophic endometrium. A) Endometrial mitochondrial OCR is decreased at baseline (EM, p = 0.02), B) energy state 3 (p = 0.03), and C) energy state 4o (p = 0.02). D) EM complex II-mediated OCR was no longer significant compared to normal endometrium (nEM) in state 3u (p = 0.04) but was significantly decreased compared to endometriosis tissue (EMO, p = 0.03). One-way ANOVA or nonparametric equivalent, p < 0.05 considered significant. (DOCX 110 kb) [file 12958_2019_513_MOESM4_ESM.docx]
