# Supplementary material for: Endometrium and endometriosis tissue mitochondrial energy metabolism in a nonhuman primate model
Source: Reprod Biol Endocrinol. 2019 Aug 24;17:70. doi: 10.1186/s12958-019-0513-8 (PMC6708555; doi:10.1186/s12958-019-0513-8)
Supplement: Supplementary file 5 — Table S1. M/z Transitions and Collision Energies (PDF 53 kb) [file 12958_2019_513_MOESM5_ESM.pdf]

Supplemental Table 1. M/z Transitions and Collision Energies

Metabolite	m/z(1)	m/z(2)	Polarity	Target Collision Energy
Glutathione	308	84.05	+	-24
NADH	666	649.1	+	-17
Glycine	75.9	30.1	+	-12
Aspartic acid	134	74.05	+	-15
Glutamine	147.1	84.05	+	-25
Lysine	147	84.1	+	-25
Threonine	120.1	74.1	+	-12
L-a-Glycerophosphocholine	257.7	104.1	+	-16
Glutamic acid	147.9	84.1	+	-20
Alanine	89.9	44.1	+	-13
Histidine	155.9	110.05	+	-20
Arginine	175.1	70.1	+	-30
Creatine	132.1	90.1	+	-14
Valine	118.1	72.1	+	-16
Methionine	149.9	56.1	+	-20
Nicotinic acid	124.1	78.05	+	-22
Leucine	132.1	86.15	+	-15
Adenosine	268.1	136.05	+	-25
FAD	786.2	136.1	+	-47
Phenylalanine	166.1	120.1	+	-20
Tryptophan	205.1	188.15	+	-10
Uracil	113	70	+	-17
Carnitine	162.1	103.05	+	-18
Glucose 6-phosphate_N	259	97	-	15
Citric acid_N	191	111.15	-	12
Malic Acid	133.1	114.95	-	17
Lactic Acid	89.3	89.05	-	7
Pyruvate	86.9	87.05	-	7
Fumaric Acid	115.1	71	-	0
MES	196.2	100.05	+	-23
